# Analysis of the Occurrence of Predicative Factors of Chronic Fatigue in Female Patients with Cancer of the Reproductive Organs with Respect to Stage of Treatment

**DOI:** 10.3390/ijerph20043732

**Published:** 2023-02-20

**Authors:** Magdalena Kłysiak, Sylwia Wieder-Huszla, Dorota Branecka-Woźniak, Katarzyna Karakiewicz-Krawczyk, Izabela Napieracz-Trzosek, Joanna Owsianowska, Anna Jurczak, Aneta Cymbaluk-Płoska

**Affiliations:** 1 Department of Gynecological Surgery and Gynecological Oncology of Adults and Adolescents, Pomeranian Medical University in Szczecin, Powstańców Wielkopolskich 72, 70-111 Szczecin, Poland; 2 Department of Clinical Nursing, Pomeranian Medical University in Szczecin, Żołnierska 48, 71-210 Szczecin, Poland; 3 Department of Gynecology and Reproductive Health Pomeranian Medical University of Szczecin, Żołnierska 48, 71-210 Szczecin, Poland

**Keywords:** cytokines, ovarian cancer, endometrial cancer, chemotherapy, women’s health

## Abstract

The aim of this study was to search for mechanisms contributing to cancer-related fatigue in patients with gynecologic cancer. The study involved 51 women with advanced endometrial cancer and ovarian cancer undergoing chemotherapy. Data were gathered at four points in time. After giving consent, each of the women had their blood drawn several times (before surgery and the first, third, and sixth cycle of chemotherapy) to determine serum levels of pro- and anti-inflammatory cytokines. Empirical data were collected using the MFSI-SF and an original questionnaire. Cancer-related fatigue (CRF) was present at every stage of treatment, but the highest mean scores were noted before cytoreductive surgery (8.745 ± 4.599), and before the sixth cycle of chemotherapy (9.667 ± 4.493). Statistically significant relationships were found between IL-1α, IL-1β, IL-2, Il-6, and IL-10 and fatigue at different stages of treatment. Older age and an above-normal BMI were the major prerequisite factors for the occurrence of fatigue in female oncological patients. The analysis of changes in cytokine levels and the severity of fatigue may be used to improve our understanding of cancer-related fatigue, and to take action to alleviate the obtrusive symptoms experienced by female patients with cancer of the reproductive organs.

## 1. Introduction

Many symptoms occur during the course of cancer, starting with pain, through nausea/vomiting and loss of appetite, to weakness. One of the most frequent symptoms experienced by oncological patients is weakness—one of the symptoms of cancer-related fatigue (CRF) [1,2]. The consequences of chronic fatigue are serious, because it affects not only the physical quality of life, but also the psychological and the social. The syndrome causes over 80% of cancer patients to reduce their daily activity, because even the simplest tasks become too difficult for them to perform [2]. The fatigue also impairs concentration, making it even more difficult to function. Patients often treat fatigue as a natural result of cancer and are not aware of the presence of other symptoms of chronic fatigue [3]. According to the literature, between 70% and 100% of patients who have undergone chemotherapy, immunotherapy, radiotherapy, or surgical treatment will have suffered from CRF, and the occurrence of the syndrome is not limited to those in poor general condition. It is often found in young patients, especially women (75%). Symptoms of CRF are also found in patients in good and very good general condition [3,4,5]. First symptoms of CRF are usually observable with the commencement of cancer treatment, although they may begin to appear during the diagnosis period, since even 50% of patients feel fatigued during diagnostic examinations aimed at determining the final diagnosis [6]. CRF symptoms usually become more severe during chemotherapy, and the occurrence of the symptoms is determined by more intense cancer treatment, previous intensity of the symptoms, and occurrence of new symptoms (e.g., pain, nausea, vomiting). Patients who undergo chemotherapy experience more intense symptoms once they receive an injection with chemotherapeutics. The symptoms are the most intense 48 to 72 h later, and their intensity depletes over the next 3 weeks. The intensity depends on the kind of chemotherapeutics that are administered [7]. Finishing the cancer treatment does not always lead to the symptoms’ quick disappearance—almost 40% of patients feel fatigued up to 3 years after they have finished chemotherapy [3]. Most patients consider chronic fatigue to be the most unpleasant symptom they experience as a result of cancer and/or oncological treatment. All symptoms resulting from CRF have negative effects on the quality of life and make physical, mental, and social functioning more difficult, frequently leading the patient to give up on treatment due to them feeling helpless, lonely, experiencing cognitive difficulties, and loosening of relationships [8]. Some patients think that fatigue signifies that the treatment is not working, or that the disease is advancing. The etiology of CRF is multi-factorial and most likely has to do with imbalances in three interconnected systems: physiological, biochemical, and psychological. Researchers have put forward numerous hypotheses regarding chronic fatigue etiology, emphasizing genetic, immunological, psychological, hormonal, and even viral causes [9,10]. The syndrome is, then, a disorder with a complex, not yet fully understood etiology, but it is apparent that advancing cancer as well as cancer treatment are factors contributing to its emergence. The causes are frequently being attributed to changes in the immune system which occur during cancer. Many studies show that patients with CRF have increased production of pro-inflammatory cytokines and an overly activated immunological response, leading to a prolonged lymphocyte activation [11,12,13,14]. IL1α, IL1β, IL2, IL5, IL6, IL8, IL10, IL13, INFγ, TNFα, and TGFβ are some of the cytokines considered to be markers of chronic fatigue syndrome [15]. Research has also confirmed disturbed functioning of cells of the immune system [8]. Therefore, observing chronic fatigue syndrome is still a challenge for researchers and clinicians, because the symptoms vary between individuals. Additionally, an incorrect interpretation of the causes induces stress, and may lead to stopping pharmacotherapy or changing the treatment plan [16]. The aim of our study was to search for mechanisms which influence the development of chronic fatigue syndrome in female patients with cancer of the reproductive organs.

## 2. Materials and Methods

### 2.1. Study Sample Selection

The study sample consisted of 51 women undergoing treatment at the Department of Gynecological Surgery and Gynecological Oncology of Adults and Adolescents of the Pomeranian Medical University in Szczecin. A prerequisite for participation was giving informed consent. The study was conducted in accordance with the Declaration of Helsinki, and the protocol was approved by the Bioethics Committee (KB-0012/81/18). The study sample included female patients with ovarian cancer and endometrial cancer who were undergoing first-line chemotherapy, or systemic treatment due to a recurrence of cancer. The patients with primary ovarian cancer underwent surgical treatment followed by 6 cycles of chemotherapy using platinum-based regimens and paclitaxel, or, if the surgical treatment was incomplete, the patients were given 18 doses of bevacizumab. In the case of a recurrence, the choice of chemotherapy depended on the platinum-sensitivity of the tumor. The patients with endometrial cancer underwent surgical treatment followed by chemotherapy and radiotherapy. The chemotherapy consisted of platinum-based regimens and paclitaxel administered in 6 cycles. In the case of recurrences, doxorubicin regimens were administered.

### 2.2. Study Design

The research procedure was divided into two parts: structured interview and biochemical analysis of the researched parameters in the serum.

#### 2.2.1. Structured Interview

The diagnostic survey method with the questionnaire technique was applied, and the standardized research tool were used to collect the empirical data: Multidimensional Fatigue Symptom Inventory-Short Form (MFSI-SF). An original survey questionnaire was also used, containing the basic sociodemographic data, that is age, place of residence, professional activity, education, marital status, menstruation, history of cancer in the family, medication administered, and physical activity.

Multidimensional Fatigue Symptom Inventory-Short Form (MFSI-SF) by Stein et al. [16,17]—this questionnaire is used to measure fatigue. It consists of 30 self-report statements relating to the last 7 days. The accuracy of each statement is evaluated on a 5-point Likert scale, from 0 to 4 (from “not at all” to “extremely”). The final score allows us to evaluate fatigue in five dimensions: General, Physical, Emotional, Mental, and Vigor. The higher the score, the more intense the fatigue, except for the Vigor scale, where the higher the score the less intense the fatigue (meaning more Vigor). The total is calculated by subtracting the points for Vigor from the subtotal of all the other scales. It is contained between -24 and 96 points. MFSI-SF does not have a set cut-off point, a high total means more intense fatigue.

#### 2.2.2. Determination of Biochemical Parameters

In accordance with the study protocol, each of the studied women had to give their consent to participate in the study. After that they had their blood drawn several times (before surgery, and before the first, third, and sixth cycle of chemotherapy). The blood was drawn on an empty stomach (at least 8 hours since the last meal), and 5.5 mL maximum of venous blood was drawn using the S-Monovette system. After the biological material was obtained, the blood was centrifugated and the serum was frozen at −80 °C until biochemical analysis could be performed.

The determination of biochemical parameters (see Supplementary Material) was performed at a certified laboratory of the Pomeranian Medical University in Szczecin using commercial, standardized methods.

The obtained serum was used to determine the concentrations of cytokines (IL-1α, IL-1β, IL-2, IL-6, TNFα, INF-γ, IL-4, IL-10). The concentrations of cytokines and analysis were performed using commercially available reagents (High Sensitivity Human ELISA kit) and an ELx800 absorbance microplate reader manufactured by BIO-TEK Instruments (Winooski, VT, USA) using wavelengths recommended by the manufacturer.

### 2.3. Statistical Analysis

Statistical analysis was performed in MedCalc software (version 20.210; Ostend, Belgium). The probability of *p* < 0.05 was considered statistically significant.

The normality of continuous variables was verified by means of Shapiro–Wilk test. Consequently non parametric Kruskal–Wallis test was used to see the differences between independent groups. Post hoc analyses were conducted by means of the Dunn method. Correlation analyses were conducted by means of Spearman’s method. For qualitative variables comparisons, the Chi square test was used. Descriptive statistics were presented as medians and interquartile ranges. 

## 3. Results

### Characteristics of the Subject Group

The subjects included in the study were 51 women diagnosed with uterine cancer (*n* = 25, 49.0%) or ovarian cancer (*n* = 26, 51.0%). The average age of the studied women equaled 61 (12 ± 8.77) years. Most of the women had secondary (47.1%) or higher (17.6%) education, and were married (31.4%). The highest number of the studied women lived in cities—88.2%, and 45.1% received a sickness allowance. Every third woman (31.4%) was employed. None of these variables’ frequency was significantly different in terms of cancer type as presented in Table 1.

**Figure 1:**
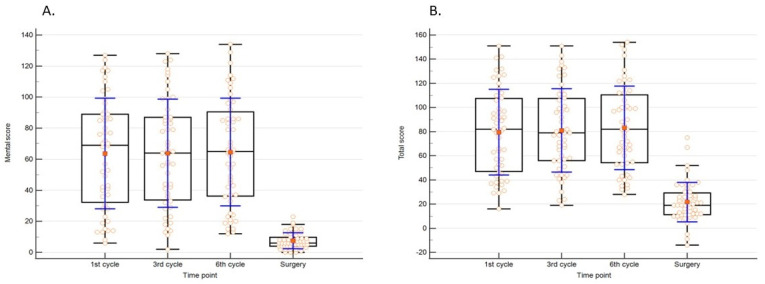
Significant differences regarding (**A**). mental and (**B**). total score in MFSI-SF in term of study points.

The studied women struggled with general fatigue at every stage of treatment, a significantly lower score for mental and overall measure (i.e., total score) was compared at the time of surgery to other time points. Data are presented in Table 2 and Figure 1.

At each time point, age did not have significant impact on any dimension measured by mean of MFSI-SF survey (Table 3).

A statistically significant dependence was found between high concentrations of all of the studied interleukins and the occurrence of all of fatigue’s dimensions in female patients with cancer of the reproductive organs at all stages of treatment. Out of the bioactive agents of the immune system, these were the concentrations of IL-6, IL-2, IL-1α, IL-1β, IL-10, TNF, IFN-γ, and IL-4 that conditioned fatigue. In the case of IL-6, it has been observed that it has a significant effect on fatigue at three of the measured points in time, both in terms of general and physical fatigue, and vigor. It also had an effect on mental fatigue at the last stage of treatment. Out of the analyzed cytokines, a statistically significant dependence was found between IL-1α, IL-1β, IL-2, Il-6, IL-10 and the occurrence of fatigue in patients at various stages of treatment. The results are presented in Table 4. 

For the total score measured by means of we found significant but moderate positive correlation with Il 1α (r = 0.407; *p* = 0.021), Il 1β (r = 0.298, *p* = 0.043), Il2 (r = 0.368; *p* = 0.049), IL 6 (r = 0.461; *p* = 0.034) and IL10 (r = 0.515, *p* = 0.002)—Table 5. 

## 4. Discussion

Advancements in cancer treatment aim at decreasing patients’ suffering resulting from the underlying disease, and at reducing treatment-related side effects. Sadly, despite these efforts and continuous advancements in medicine, cancer patients still complain about having a poor quality of life due to treatment. One of the most frequently mentioned side effects is weakness and/or fatigue. CRF, which accompanies cancer, is defined as a prolonged, debilitating, and subjective feeling of fatigue which affects cancer patients during illness and treatment, therefore it is a result of the disease or applied treatment [18,19]. Apart from fatigue, patients complain about becoming tired quickly, loss of sleep or feeling overly sleepy, being in a bad mood, and having trouble concentrating. Problems such as these often lead to emotional distress and feeling helpless and lonely. Unlike fatigue in healthy people, these symptoms found in cancer patients do not go away after rest and can affect up to 80–90% of patients undergoing treatment, or in advanced stages of cancer [20,21,22].

According to Brown et al., cancer patients who experience more fatigue function physically worse compared to others [23]. De Jong’s team, based on a literature review, found that fatigue is one of the most common side effects of chemotherapy, since high and fluctuating indicators of fatigue are found both during treatment and after it has finished [24]. In our own study, the patients suffered from general fatigue at all stages of treatment, with slightly higher levels of fatigue before cytoreductive surgery and the sixth cycle of chemotherapy. When we compared the dimensions of fatigue at each measured point in time, we observed that the mean score was the highest for mental fatigue in patients before surgical treatment, and for physical fatigue in patients before the sixth cycle of chemotherapy. At the same time, patients entering the last stage of treatment had high mean scores for vigor. Our results may confirm that the fatigue experienced by patients at the initial stages of illness is due to the diagnosis and diagnostic examinations aimed at determining the final diagnosis [6]. The symptoms of chronic fatigue increase during chemotherapy, reaching a critical point before it finishes, but approaching the end of treatment gives the body new energy and a fighting spirit.

Studies by Maurer’s team showed that the level of education, BMI, level of physical activity, and chronic inflammatory diseases present prior to the diagnosis are strong predicators of long-term CRF. According to the researchers, lifestyle, coexisting conditions, and socioeconomic factors may be more informative in identifying patients at risk of long-term CRF than the presence of inflammation biomarkers [5]. Bower et al., on the other hand, have analyzed the effects of chronic illnesses on the level of fatigue. Their results confirmed that women showing symptoms of fatigue are more likely to suffer from diabetes, arterial hypertension, or heart conditions [25]. Our own studies confirm the influence of sociodemographic factors, i.e., age, education, professional activity, as well as of medical factors, i.e., BMI and co-existence of diabetes, on the feelings of fatigue in all its dimensions.

Many studies have shown that cancer treatment causes imbalances in the immune system resulting in long-term inflammation. Higher concentrations of pro-inflammatory cytokines are symptomatic of that [26,27]. Searches for biomarkers of cancers of the reproductive organs reaffirm the effects of interleukins in the development of these cancers. TNF-α, IL-1, and IL-6 participate in the development of endometriosis, and carcinogenesis of the endometrium [28,29]. The literature also confirms the participation of IL-6 as an inflammatory factor in the proliferation of cancerous cells in some cancers [30,31], including metastasis of uterine cancer [32,33]. A high concentration of IL-6 in the serum of endometrial cancer patients is linked both with the carcinogenesis of the endometrium [34,35,36,37] and with advancement of the cancer [34]. High concentrations of cytokines IL-4, IL-6, and IL-10 are also found in cases of ovarian cancer [38,39], and studies by Clendenen et al. provide proof that high concentrations of IL-2, IL-4, and IL-6 are closely tied to the risk of developing this type of cancer [40]. According to the researchers, topical inflammatory cytokines such as IL-1β and TNF-α are the main inductors of the expression and secretion of IL-6 [41,42]. High concentrations of IL-1β and TNF-α have also been found in the serum of patients with advanced ovarian cancer [43,44,45,46]. It has also been shown that the high concentration of TNFα found in these patients [45,47] correlates strongly with increased advancement of the cancer [45,47], a shortened survival period [48], and increased expression of IL-6 [49]. Inflammatory factors can modulate the response of cancerous cells to chemotherapy, and anti-cancer drugs can cause the expression of some of the cytokine genes, including those of TNFα, IL-1β, and IL-6 [50,51,52]. Numerous studies have shown lower concentrations of the analyzed cytokines in the serum of patients treated with chemotherapy [45,53,54].

According to Panju et al. and Inagaki et al., changes in the concentrations of cytokines, especially IL-6, IL-1β, and TNF-α, may lead to sickness behaviors, including symptoms of fatigue [55,56]. Collado-Hidalgo’s team conducted research on the dependence between markers of fatigue and inflammation, and their results showed increased production of interleukin IL-6 and TNF-α in breast cancer survivors with symptoms of fatigue [57]. Maurer et al.’s longitudinal study on breast cancer patients confirmed the dependence between IL-6 and chronic fatigue. That being said, some believe that the diagnostic value of IL-6 as a fatigue marker may be limited, due to its double effects, both pro- and anti-inflammatory [30]. This is why some researchers choose to focus on unambiguous inflammation biomarkers, such as IL-1β and TNF-α, even though Maurer’s team’s results have not shown that they have any noteworthy effects on CRF [25]. Ahlberg et al. observed an increase in fatigue in uterine cancer patients undergoing radiotherapy; however, they did not observe any noteworthy changes in the concentrations of IL-1, IL-6, or TNF-α [58]. Kwak et al. [59] and Orre’s team [60,61] also found no dependence between the level of fatigue and the pro-inflammatory cytokine IL-6. Our own studies confirm the influence of cytokines (i.e., concentrations of IL-1A, IL-1B, IL-2, IL-6, IL-10, TNF, and IL-4) on the emergence of fatigue in all its dimensions in patients with cancers of the reproductive organs at various stages of treatment.

In Goff’s team’s research, a higher concentration of IL-6 was closely tied to fatigue before surgery. Authors describe the existence of fatigue in ovarian cancer as “a pre-diagnostic symptom”, because it is one of the most frequently occurring symptoms patients complain about before they are diagnosed with this type of cancer [62,63]. In Inagaki’s team’s research, the concentration of IL-6 correlated strongly with physical fatigue in patients with terminal cancer [55]. In our own studies, IL-6 had a significant effect on the symptoms of fatigue both before surgery, as well as before the first and sixth cycle of chemotherapy, not only in the physical dimension, but also in the general dimension and vigor. The relationship between fatigue and the concentration of IL-6 before surgery was also observed by Clevenger’s team [64]. Inflammatory markers may be direct causes of fatigue, because they affect pathways of the central nervous system, causing vegetative behaviors [57,65,66,67,68,69,70]. Redeker observed that higher concentrations of IL-6 before surgery correlated significantly with sleep disturbances and tiredness, and the lowering of the cytokine’s concentration between the surgery and a year from it had to do with a better quality of sleep and decrease in fatigue [71].

When conducting a systematic research review, Salignan’s team proved that increased symptoms of fatigue, especially in women in early stages of breast cancer, were caused by increased concentrations of IL-6 and TNF, and of IL-1β during chemotherapy, and of IL-6 during radiotherapy [72]. On the other hand, Panju et al.’s studies on patients with acute myeloid leukemia showed strong correlations between fatigue and IL-10 between the tested time periods—the beginning and after 4–6 weeks [56]. In our own studies, IL-1β correlated with fatigue at all of the measured points in time, determining vigor and emotional fatigue before cytoreductive surgery, and physical fatigue before the sixth cycle of chemotherapy. TNF and IL-10, on the other hand, affected not only general fatigue and vigor before surgery, but also mental fatigue before the third cycle of chemotherapy, and determined general and mental fatigue and vigor before the end of treatment.

The causes of cancer-related chronic fatigue have not yet been determined, but researchers agree that the phenomenon is multifactorial. Studies of inflammation biomarkers are justified, because they facilitate a better understanding of the biological pathways related to CRF, and achieving a better therapeutic effect whilst preserving a high quality of life of patients.

Our study has some limitations. First of all, a limited number of participants were screened at each study phase. In addition, we did not intend to compare the results with controls, thus we did not recruit either healthy women (matched for age and sex) or patients with benign conditions such as, for instance, endometriosis. Additionally, comparing the observed results with ones collected in women undergoing other surgical procedures (e.g. laparoscopy; robotic surgery) could act as an added value to the present research. Further research on such comparisons is warranted.

## 5. Conclusions

We observed the dependence between the concentrations of selected cytokines and fatigue in the studied group of patients. The dominating factors predisposing the occurrence of fatigue in the patients were age, higher than normal BMI, and, to a smaller degree, education, professional activity, and diabetes. Analyzing the changes in cytokine concentrations and fatigue intensity (in all its dimensions) may allow us to better understand the mechanisms behind cancer-related fatigue. Evaluating how these components change during treatment may help identify the kind of interventions which will help alleviate the most debilitating symptoms in patients with cancer of the reproductive organs.

## Figures and Tables

**Table 1:** Sociodemographic data depending on cancer type.

		Cancer Type		*p*
		Ovarian Cancer	Uterine Cancer	
Education	primary	3	1	0.72
	vocational	5	5	
	secondary	13	12	
	higher	5	7	
Place of Residence	village	4	2	0.59
	city ≤ 10k inhabitants	9	6	
	city ≤ 100k inhabitants	7	8	
	city > 100k inhabitants	6	9	
Marital Status	single	3	2	0.66
	married	14	10	
	widow	7	10	
	divorced	2	3	
Working Activity	working	8	9	0.1
	not working	9	3	
	pension	9	10	
	sickness pension	0	3	
Hypertension	no	16	10	0.1
	yes	10	15	
Diabetes	no	14	14	0.55
	yes	12	11	
Thyroid Disease	no	14	14	0.59
	yes	12	11	
BMI value	above normal	14	16	0.32
	normal	12	9	

*p*—probability.

**Table 2:** Occurrence and intensity of fatigue depending on stage of treatment.

General Score						
Factor	Minimum	25th Percentile	Median	75th Percentile	Maximum	*p*
1ST CYCLE	0	4	5	9	16	0.31
3RD CYCLE	0	3	5	10	22	
6TH CYCLE	0	4.25	7	11	22	
SURGERY	0	5	6	8.75	23	
Emotional score						
1ST CYCLE	0	5	9	11	15	0.16
3RD CYCLE	1	6	9	11	22	
6TH CYCLE	4	8	9	11	22	
SURGERY	0	5	8	10.75	22	
Mental score						
1ST CYCLE	6	32.25	69	89	127	<0.05 *
3RD CYCLE	2	33.75	64	87	128	
6TH CYCLE	12	36.25	65	90.5	134	
SURGERY	0	4	6	9.75	23	
Physical score						
1ST CYCLE	2	6	7	10	17	0.07
3RD CYCLE	0	4.25	7	11	24	
6TH CYCLE	3	6	9	11.75	24	
SURGERY	0	4	7	9	22	
Vigor						
1ST CYCLE	0	3.25	6	9.75	22	0.1
3RD CYCLE	0	2	6	10	20	
6TH CYCLE	0	5	8	11	20	
SURGERY	1	5	8	11.75	22	
Total score						
1ST CYCLE	16	47	82	107.5	151	<0.05 *
3RD CYCLE	19	56	79	107.5	151	
6TH CYCLE	28	54.25	82	110.5	154	
SURGERY	−14	11.25	19	29.25	75	

* *p* < 0.05 (statistically significant).

**Table 3:** Influence of age on fatigue according to MFSI-SF.

Dimension		Surgery	1st Cycle	3rd Cycle	6th Cycle
Emotional	Correlation coefficient	0.149	0. 189	0.145	0.053
	Significance Level *p*	0.2955	0.1841	0.3108	0.7126
General	Correlation coefficient	−0.044	−0.049	0.133	0.144
	Significance Level *p*	0.7584	0.7343	0.3512	0.3144
Mental	Correlation coefficient	0.148	0.117	0.127	0.125
	Significance Level *p*	0.3004	0.4127	0.3733	0.3812
Physical	Correlation coefficient	0.006	0.001	0.08	0.04
	Significance Level *p*	0.9653	0.9968	0.5784	0.7802
Total	Correlation coefficient	0.043	0.118	0.121	0.077
	Significance Level *p*	0.7646	0.4091	0.3986	0.5902
Vigor	Correlation coefficient	−0.022	0.136	0.003	0.181
	Significance Level *p*	0.877	0.3412	0.983	0.2028

*p*—probability.

**Table 4:** Measured elements depending on stage of treatment and occurrence of fatigue in its dimensions.

Dimensions of Fatigue	Surgery	1st Cycle	3rd Cycle	6th Cycle
TNF-γ				
General	R = 0.246; *p* = 0.368	R = 0.323; *p* = 0.123	R = 0.368; *p* = 0.068	R = 0.411; *p* = 0.032
Physical	R = 0.404; *p* = 0.031	R = 0.378; *p* = 0.256	R = 0.299; *p* = 0.114	R = 0.322; *p* = 0.108
Emotional	R = 0.328; *p* = 0.066	R = 0.402; p = 0.215	R = 0.371; *p* = 0.308	R = 0.355; *p* = 0.067
Mental	R = 0.428; *p* = 0.171	R = 0.368; *p* = 0.152	R = 0.399; *p* = 0.045	R = 0.422; *p* = 0.026
Vigor	R = 0.388; *p* = 0.040	R = 0.404; *p* = 0.217	R = 0.392; *p* = 0.321	R = 0.402; *p* = 0.049
IL-1 α				
General	R = 0.496; *p* = 0.037	R = 0.421; *p* = 0.131	R = 0.401; p = 0.107	R = 0.392; *p* = 0.089
Physical	R = 0.502; *p* = 0.146	R = 0.562; *p* = 0.042	R = 0.497; p = 0.101	R = 0.484; *p* = 0.218
Emotional	R = 0.423; *p* = 0.022	R = 0.361; *p* = 0.224	R = 0.369; p = 0.301	R = 0.412; *p* = 0.013
Mental	R = 0.395; *p* = 0.071	R = 0.406; *p* = 0.342	R = 0.413; p = 0.073	R = 0.441; *p* = 0.058
Vigor	R = 0.526; *p* = 0.030	R = 0.542; *p* = 0.187	R = 0.505; p = 0.217	R = 0.498; *p* = 0.016
IL-1β				
General	R = 0.391; *p* = 0.228	R = 0.296; *p* = 0.223	R = 0.232; *p* = 0.152	R = 0.327; *p* = 0.204
Physical	R = 0.414; *p* = 0.109	R = 0.408; *p* = 0.135	R = 0.393; *p* = 0.126	R = 0.344; *p* = 0.015
Emotional	R = 0.326; *p* = 0.041	R = 0.412; *p* = 0.362	R = 0.322; *p* = 0.114	R = 0.361; *p* = 0.189
Mental	R = 0.196; *p* = 0.213	R = 0.152; *p* = 0.216	R = 0.201; *p* = 0.178	R = 0.162; *p* = 0.048
Vigor	R = 0.298; *p* = 0.040	R = 0.313; *p* = 0.034	R = 0.274; *p* = 0.031	R = 0.336; *p* = 0.128
IL-2				
General	R = 0.427; *p* = 0.046	R = 0.458; *p* = 0.069	R = 0.412; *p* = 0.072	R = 0.442; *p* = 0.004
Physical	R = 0.381; *p* = 0.008	R = 0.513; *p* = 0.164	R = 0.602; *p* = 0.027	R = 0.621; *p* = 0.093
Emotional	R = 0.248; *p* = 0.122	R = 0.326; *p* = 0.061	R = 0.367; *p* = 0.162	R = 0.394; *p* = 0.009
Mental	R = 0.301; *p* = 0.372	R = 0.348; *p* = 0.723	R = 0.391; *p* = 0.607	R = 0.325; *p* = 0.055
Vigor	R = 0.612; *p* = 0.022	R = 0.517; *p* = 0.671	R = 0.496; *p* = 0.185	R = 0.522; *p* = 0.038
IL-6				
General	R = 0.501; *p* = 0.023	R = 0.583; *p* = 0.047	R = 0.528; *p* = 0.195	R = 0.515; *p* = 0.026
Physical	R = 0.499; *p* = 0.036	R = 0.602; *p* = 0.082	R = 0.537; *p* = 0.174	R = 0.525; *p* = 0.027
Emotional	R = 0.485; *p* = 0.624	R = 0.422; *p* = 0.096	R = 0.418; *p* = 0.059	R = 0.464; *p* = 0.365
Mental	R = 0.323; *p* = 0.711	R = 0.458; *p* = 0.527	R = 0.462; *p* = 0.129	R = 0.529; *p* = 0.046
Vigor	R = 0.459; *p* = 0.037	R = 0.427; *p* = 0.288	R = 0.399; *p* = 0.294	R = 0.503; *p* = 0.041
IL-4				
General	R = 0.261; *p* = 0.227	R = 0.211; *p* = 0.384	R = 0.277; *p* = 0.402	R = 0.232; *p* = 0.322
Physical	R = 0.301; *p* = 0.288	R = 0.341; *p* = 0.164	R = 0.233; *p* = 0.032	R = 0.269; *p* = 0.129
Emotional	R = 0.224; *p* = 0.422	R = 0.183; *p* = 0.075	R = 0.411; *p* = 0.121	R = 0.327; *p* = 0.262
Mental	R = 0.266; *p* = 0.004	R = 0.292; *p* = 0.415	R = 0.261; *p* = 0.066	R = 0.274; *p* = 0.133
Vigor	R = 0.227; *p* = 0.373	R = 0.295; *p* = 0.622	R = 0.267; *p* = 0.381	R = 0.219; *p* = 0.029
IL-10				
General	R = 0.555; *p* = 0.304	R = 0.446; *p* = 0.312	R = 0.501; *p* = 0.166	R = 0.492; *p* = 0.021
Physical	R = 0.660; *p* = 0.012	R = 0.489; *p* = 0.277	R = 0.515; *p* = 0.159	R = 0.412; *p* = 0.229
Emotional	R = 0.494; *p* = 0.604	R = 0.361; *p* = 0.316	R = 0.383; *p* = 0.426	R = 0.418; *p* = 0.743
Mental	R = 0.451; *p* = 0.231	R = 0.488; *p* = 0.192	R = 0.426; *p* = 0.026	R = 0.399; *p* = 0.003
Vigor	R = 0.723; *p* = 0.044	R = 0.426; *p* = 0.175	R = 0.394; *p* = 0.273	R = 0.483; *p* = 0.011
IFN-gamma				
General	R = 0.387; *p* = 0.102	R = 0.317; *p* = 0.069	R = 0.442; *p* = 0.022	R = 0.513; *p* = 0.066
Physical	R = 0.660; *p* = 0.006	R = 0.551; *p* = 0.140	R = 0.603; *p* = 0.184	R = 0.482; *p* = 0.192
Emotional	R = 0.262; *p* = 0.385	R = 0.402; *p* = 0.190	R = 0.447; *p* = 0.201	R = 0.425; *p* = 0.046
Mental	R = 0.388; *p* = 0.424	R = 0.398; *p* = 0.384	R = 0.444; *p* = 0.177	R = 0.408; *p* = 0.132
Vigor	R = 0.412; *p* = 0.248	R = 0.621; *p* = 0.062	R = 0.503; *p* = 0.296	R = 0.526; *p* = 0.001

R—correlation coefficient, *p*—probability.

**Table 5:** Correlation results by means of Spearman’s method.

Il 1α	Il 1β	Il2	IL 6	IL10
R = 0.407 *p* = 0.021	R = 0.298 *p* = 0.043	R = 0.368 *p* = 0.049	R = 0.461 *p* = 0.034	R = 0.515 *p* = 0.002

## Data Availability

The data presented in this study are available on request from the first author.

## Supplementary Materials

The following supporting information can be downloaded at: https://www.mdpi.com/article/10.3390/ijerph20043732/s1, Table S1: Data on measured biochemical parameters in each study time.
